# Knowledge and practice among caregivers having children with autism in Bangladesh: findings from a cross-sectional study

**DOI:** 10.1186/s13104-024-07074-2

**Published:** 2025-02-24

**Authors:** Md. Al Imran, Md. Saiful Islam, Md. Sharif Hossain, Shahina Pardhan, Nakiba Bari, Zebunnesa Zeba

**Affiliations:** 1 Department of Public Health and Informatics, Jahangirnagar University, Savar, Dhaka, 1342 Bangladesh; 2 Centre for Advanced Research Excellence in Public Health, Savar, Dhaka, 1342 Bangladesh; 3 Vision and Eye Research Institute, School of Medicine, Anglia Ruskin University, Young Street, Cambridge, UK; 4 Knowledge Management, Gender Justice and Diversity, BRAC, Dhaka, 1212 Bangladesh; 5 Department of Special Education, University of Dhaka, Dhaka, 1000 Bangladesh; 6 Division of Epidemiology, Biostatistics, and Environmental Health, School of Public Health, The University of Memphis, Memphis, Tennessee 38152-3330 United States

**Keywords:** Knowledge, Practice, Autistic, Children, Bangladesh, ASD

## Abstract

**Background:**

Autism spectrum disorder (ASD) is a term used to describe a group of conditions characterized by difficulties with social skills, speech, repetitive behaviors, and nonverbal communication. There is no cure for autism, however, early diagnosis and intervention can increase the chance of treatment success. If parents or caregivers do not have sound knowledge about autism, problems can become more complicated. The study aimed to assess the knowledge and practice among caregivers having children with ASD in Bangladesh.

**Methods:**

A cross-sectional survey was conducted among 68 caregivers of children with ASD in the selected area of Mymensingh city, Bangladesh. The data were collected from May to June 2021 through face-to-face interviews by a semi-structured questionnaire including informed consent, socio-demographics, as well as questions regarding knowledge (12-item) and practice (6-item) towards children with ASD using a purposive sampling technique. The data were analyzed using the SPSS software (version 25.0).

**Results:**

The mean score of knowledge among caregivers having children with ASD was 7.16 (SD = 2.09) out of 12 (59.67%). The mean score of practice among caregivers having children with ASD was 3.16 (SD = 1.10) out of 6 (52.67%). There were no significant mean differences in the mean knowledge and practice scores among participants’ different socio-demographic categories. 95.6% of caregivers have not received any formal training to care for children with ASD, and more than half (57.4%) believed that mixing with good friends would make the necessary change in children with ASD. 97.1% of the children with ASD did not have any health insurance with 72.1% receiving government allowance for ASD.

**Conclusions:**

The findings indicated inadequate knowledge and practice among caregivers of children with ASD. The study suggests an immediate health education program is needed, as well as appropriate practice for children with ASD in Bangladesh.

## Introduction

Autism Spectrum Disorder (ASD) is a neurological disorder that is caused by abnormal biology and the chemistry of the brain [1, 2], which could be due to genetic changes [3]. If a family has a history of autism, then children in that family are much more likely to have autism [4]. Autism is caused by a combination of infections, metabolic, genetic, and environmental factors [5]. Additionally, maternal rubella, tuberoses, and encephalitis, which are conditions that affect brain development, may be linked to autism [6]. The etiology of autism is complex and could also be influenced by environmental factors [7]. Autism spectrum disorders (ASDs) have been linked to some sociodemographic factors such as maternal and paternal age, race, and socioeconomic status. Furthermore, women over the age of 40 have a 50% greater risk of having a child with autism than women between the ages of 20 and 29 [8].

According to the DSM-IV-TR, various conditions for ASD include childhood autism, childhood disintegrative disorder, Rett’s syndrome, Asperger’s syndrome, and pervasive developmental disorders – not otherwise specified. Baxter et al. found a global prevalence of 7.6 per 1000, or one in 132 people [11]. ASD is estimated to affect one in 160 children in Southeast Asia [9, 10]. Despite the fact that the South Asia region (i.e., Bangladesh, India, Pakistan, Nepal, Sri Lanka, Bhutan, Maldives, and Afghanistan) accounts for more than 20% of the world’s population, the prevalence of ASD in this region is still mostly unidentified [9, 13]. Bangabandhu Sheikh Mujib Medical University (BSMMU) recently reported that nearly 1 in 500 children in Bangladesh have ASD [12], with a higher urban prevalence than in rural areas [12].

The first three years of life are crucial, as this is the period in a child’s life when their brain is most malleable and experiences rapid growth [14]. A child exposed to neglect or violence may suffer cognitive, behavioral or emotional difficulties [15]. In a family with ASD children, caregivers face a variety of challenges, including decreased parenting efficacy, increased parenting stress, and an increase in mental and physical health issues. High rates of divorce and lower overall family well-being, plus significant financial strain and time pressures have been reported in families who have a child with ASD [16]. It may be possible that these have a negative impact on the diagnosed child and can even counteract the positive effects of any positive effects of intervention.

The economic landscape may make the care required for the children more difficult, especially with mothers working in urban factories who would need to balance their responsibilities. In addition, the majority of city workers are migrants from rural areas, who might be separated from their children living back at home. Mothers of children with autism receive insufficient assistance from their husbands or other family members, in rearing children with autism in Bangladesh [17]. Reports suggest that children with autism are admitted to school but are then forced to discontinue after complaints from other students [18].

Lack of parental awareness would be a major deterrent in any support that the family and the child may be able to obtain. It is possible that parents may hesitant or unwilling to treat children with ASD until it is too late [19]. As a result, it is possible that an autistic child born into these families is kept apart from all social contacts leading to isolation [20].

There is limited research about knowledge and practice among caregivers having children with ASD in Bangladesh which this study aims to explore.

## Methods

### Study design, setting, and participants

A descriptive type of cross-sectional survey was conducted among 68 caregivers of children with ASD in the selected area of Mymensingh City, Bangladesh. The participants were recruited using a purposive sampling technique. The data were collected through face-to-face interviews between May and June 2021. Inclusion criteria were being parents and caregivers of children with ASD and being adults (≥ 18 years). Those who had language or speech difficulties, and caregivers of children with ASD who had recently been diagnosed with any illness, such as physical or severe psychological illness, were excluded.

### Study instrument

A semi-structured questionnaire including four sections was used to collect data. The questionnaire was adopted from the previous literature [21–24]. Section one included the socio-demographic information of the caregivers and children with ASD; section two investigated the level of knowledge of caregivers about children with ASD; section three was the ways of practice towards children with ASD; and section four was available services for the children with ASD.

### Socio-demographic information

Socio-demographic information was asked during the interview periods including age of ASD children, caregiver relationship with ASD children, sex of ASD children, educational level of caregiver, number of family members, family type, occupation, monthly family income, and family history of ASD. Later monthly family income was classified into the following categories: < 5000 Bangladeshi Taka (BDT), 5000–10,000 BDT, 10,001–15,000 BDT, 15,001–20,000 BDT, and > 20,000 BDT. 1 USD ≈ 109.63 BDT (as of 26 March 2024).

### Knowledge about ASD

The knowledge of participants was measured using twelve-item questions (e.g., *“Children with ASD have trouble communicating with others?”*; see details in Table 2). Among these, six were answered using four responses (always/ sometimes/ never/ don’t know); and the remaining six were answered using three responses (yes/ no/ sometimes). Correct responses were coded as “1”; and incorrect responses were coded as “0”. The total score was calculated by summating the raw score of each item, with higher scores indicating better level of knowledge.

### Patrice towards children with ASD

A total of six-item questions were asked to discover participants’ practice towards children with ASD (e.g., “*Have you received formal training required to care for an ASD child?”;* see details in Table 4). Favorable responses were coded as “1”; and unfavorable/ unexpected responses were coded as “0”. The total score was calculated by summating the raw score of each item, with higher scores indicating the better level of practice.

### Knowledge about available services for the children with ASD

The participants were asked about available services for the children with ASD in their community. A total of three questions were asked (*Do you have health insurance for your child?; Does your child get government allowance for ASD?;* and *How well do you think the media portrays the ASD problem?*)

### Data processing and analysis

Data were analyzed using SPSS software (version 25.0). Descriptive analysis was calculated including means and standard deviations for continuous variables; and frequencies and percentages for categorical variables. Bivariate analyses including t-test, ANOVA, and Pearson correlation were performed as appropriate to obtain the association of outcome variables (knowledge and practice) with socio-demographic variables. A *p*-value less than 0.05 was considered as statistically significant.

### Ethical approval

The study protocol was reviewed and approved by the Biosafety, Biosecurity and Ethical Clearance Committee, Jahangirnagar University, Savar, Dhaka-1342, Bangladesh [Ref No: BBEC, JU/ M- 2022/ 2 (1)]. The study was conducted in accordance with the ethical principle involving human participation (Helsinki Declaration). Before the interview, the study’s purpose and its voluntary nature of participants were explained to each participant. Prior to giving their consent, participants were informed that they could refuse to answer any questions or leave the interview at any time. The interview data were anonymous and analyzed using a coding system. No monetary or other incentives were given to the participants.

## Results

### General characteristics of the participants

A total of 68 caregivers of children with ASD were included in the final analysis. The mean age of the children that the caregivers looked after was 15.51 years (SD = 6.36) and the majority were males (57.4%) (Table 1). More than half of the caregivers were mothers (51%), 22.1% were fathers, and others (26.5%). 41.2% of participants reported they had secondary education, and 26.5% had primary education. 80.9% of the respondents belonged to a nuclear family, and 42.6% had a monthly family income ranging from 5000 to 1000 BDT. 14.7% of participants reported that had other family members with ASD.

**Table 1:** Distribution of the socio-demographic variable (*N* = 68)

Variables	Frequency	Percentage
**Age of the child with ASD**	Mean, SD = 15.51, 6.36	
**Caregiver relationship with child with ASD**		
Father	15	22.1
Mother	35	51.5
Other	18	26.5
**Sex of the child with ASD**		
Male	39	57.4
Female	29	42.6
**Educational level of the caregiver**		
No primary education	1	1.5
Primary education	18	26.5
Secondary education	28	41.2
Higher secondary education	14	20.6
Honors (undergraduate)	7	10.3
Number of family members	Mean, SD = 5.41, 1.518	
**Family type**		
Nuclear	55	80.9
Joint	13	19.1
**Occupation**		
Student	7	10.3
Govt. employee	6	8.8
Non-govt. employee	5	7.4
Businessperson	7	10.3
Stay at home parent	37	54.4
Other	6	8.8
**Monthly family income (BDT)**		
< 5000	10	14.7
5000-10000	29	42.6
10001-15000	17	25
15,001- 20,000	4	5.9
> 20,000	8	11.8
**Do any family members have ASD?**		
Yes	10	14.7
No	58	85.3

Note: BDT: Bangladeshi Taka

### Knowledge about ASD

Table 2 represents the distribution of participants’ knowledge about children with ASD. 55.9% reported that children with ASD had trouble communicating with others sometimes, 57.4% reported that the children they cared for had behavioral problems, 66.2% reported that the children responded when called by name, and 62% believed that children with ASD can communicate with others through speaking, gestures or expression.

The mean score of knowledge among caregivers was 7.16 (SD = 2.09) out of 12, or 59.67%. There was no significant mean difference in the mean knowledge score among participants’ different socio-demographic categories (Table 3).

**Table 2:** Distribution of knowledge among caregivers having children with ASD

Variables	Frequency	Percentage
**Children with ASD have trouble communicating with others?**		
Always	13	19.1
Sometimes	38	55.9
Never	13	19.1
Don’t know	4	5.9
**Children with ASD avoid eye contact?**		
Always	17	25
Sometimes	29	42.6
Never	17	25
Don’t know	5	7.4
**Children with ASD repeat the same behavior or have too much interest in something?**		
Always	21	30.9
Sometimes	28	41.2
Never	13	19.1
Don’t know	6	8.8
**Children with ASD have behavioral problems? (E.g.**,** throwing toys**,** aggression**,** shouting)**		
Always	5	7.7
Sometimes	39	57.4
Never	18	26.5
Don’t know	6	8.8
**Are children with ASD sensitive to changes in the environment? (E.g.**,** cold**,** hot)**		
Always	5	7.4
Sometimes	23	33.8
Never	23	33.8
Don’t know	17	25
**Children with ASD have any exceptional talents?**		
Always	2	2.9
Sometimes	14	20.6
Never	34	50.0
Don’t know	18	26.5
**Can ASD children better communicate desires**,** aspirations**,** and beliefs to others?**		
Yes	12	17.6
No	44	64.7
Sometimes	12	17.6
**Show any interest or get attention by using different gestures?**		
Yes	37	54.4
No	16	23.5
Sometimes	15	22.1
**Show an abnormal or variable response to sound? (E.g.**,** startled or unresponsive or overly sensitive)**		
Yes	32	47.1
No	23	33.8
Sometimes	13	19.1
**Responds when called by name?**		
Yes	45	66.2
No	18	26.5
Sometimes	5	7.4
**Shows interest in games generally?**		
Yes	36	52.9
No	21	30.9
Sometimes	11	16.2
**Able to communicate with others through speaking**,** gestures or expression?**		
Yes	42	61.8
No	13	19.1
Sometimes	13	19.1

**Table 3:** Association between socio-demographic information and parental knowledge

Variables	Mean (SD)	t/F	*p*-value
**Age of the child**		0.05*	0.714
**Relationship with child**			
Father	7.2 (1.66)	0.83	0.442
Mother	6.89 (2.00)		
Other	7.67 (2.57)		
**Sex of the ASD child**			
Male	7.18 (1.97)	0.01	0.936
Female	7.14 (2.28)		
**Educational level of the caregiver**			
No primary education	5 (0.00)	1.67	0.169
Primary	7.72 (1.64)		
Secondary	6.93 (2.43)		
Higher secondary	7.71 (1.82)		
Honors	5.86 (1.68)		
**Number of family members**		-0.02*	0.863
**Family type**			
Nuclear	7.16 (2.19)	0.00	0.988
Joint	7.15 (1.68)		
**Occupation**			
Student	6.71 (2.75)	1.06	0.393
Govt. employee	7 (1.55)		
Non-govt. employee	7.6 (2.07)		
Businessperson	8.57 (1.99)		
Stay at home parent	6.84 (2.01)		
Other	7.83 (2.32)		
**Monthly family income (BDT)**			
< 5000	6.4 (1.78)	1.52	0.207
5000-10000	7.79 (2.37)		
10001-15000	7.12 (1.58)		
15,001- 20,000	6.5 (2.38)		
> 20,000	6.25 (1.91)		
**Do any family members have ASD?**			
Yes	6.4 (1.84)	1.57	0.215
No	7.29 (2.12)		

Note: *Pearson correlation; BDT: Bangladeshi Taka

### Practice towards children with ASD

Table 4 shows that 95.6% of the caregivers had not received any formal training to care for children with ASD, more than half (57%) of them believed that mixing with good friends would change the behavior of their ASD child, and 61.8% believed that treatment could be started as soon as ASD has been diagnosed (Table 4).

**Table 4:** Practice towards children with ASD related information

Variables	Frequency	Percentage
**Have you received formal training required to care for a child with ASD?**		
Yes	3	4.4
No	65	95.6
**Did you go to the doctor when you noticed something unusual in your baby?**		
Yes	51	75
No	17	25
**Which of the following can be of great benefit to children with autism?**		
Mix with good friends	39	57.4
By keeping them under extreme compulsion	1	1.5
Don’t know	27	39.7
Other	1	1.5
**What measures do you think any child with ASD would be happy with in their life?**		
Friends and support	21	30.9
An experienced doctor	27	39.7
Family care	17	25
Other	3	4.4
**What will parents do if they think their child is showing early signs of autism?**		
Take the child to the emergency room	2	2.9
Contact a doctor as soon as possible and include regular screenings	59	86.8
Hides the child from the neighbors	7	10.3
**How soon can care be started if the symptoms of autism are seen in a child?**		
At the age of 3 years	8	11.8
At the age of 5 years	2	2.9
When parents feel the need	16	23.5
When the child is diagnosed	42	61.8

The mean score of practice among caregivers having children with ASD was 3.16 (SD = 1.10) out of 6, or 52.67%. There was no significant mean difference in the mean practice score among participants’ different socio-demographic categories (Table 5).

**Table 5:** Association between socio-demographic information and practice score

Variables	Mean (SD)	t/F	*p*-value
**Age**		0.02	0.845
**Relationship with child**			
Father	3.07 (1.1)	0.16	0.855
Mother	3.14 (1.19)		
Other	3.28 (0.96)		
**Sex of the child with ASD**			
Male	3.28 (1.15)	1.09	0.300
Female	3 (1.04)		
**Educational level of the caregiver**			
No primary education	4 (0)	0.67	0.613
Primary	3 (1.03)		
Secondary	3.04 (1.04)		
Higher secondary	3.36 (0.93)		
Honors	3.57 (1.81)		
**Family member**		0.02	0.858
**Family type**			
Nuclear	3.13 (1.02)	0.28	0.599
Joint	3.31 (1.44)		
**Occupation**			
Student	2.86 (1.07)	0.81	0.545
Govt. employee	3.17 (1.33)		
Non-govt. employee	3.2 (1.92)		
Businessperson	3.71 (1.11)		
Stay at home parent	3.03 (0.99)		
Others	3.67 (0.82)		
**Monthly family income (BDT)**			
< 5000	3.3 (0.95)	0.42	0.791
5000-10000	3 (1.1)		
10001-15000	3.41 (1.18)		
15,001- 20,000	3 (0.82)		
> 20,000	3.13 (1.36)		
**Do any family members have ASD?**			
Yes	2.9 (1.37)	0.66	0.420
No	3.21 (1.06)		

Note: *Pearson correlation; BDT: Bangladeshi Taka

**Table 6:** Distribution of services available for ASD care

Variables	Frequency	Percentage
**Do you have health insurance for your child?**		
Yes	2	2.9
No	66	97.1
**Does your child get government allowance for ASD?**		
Yes	49	72.1
No	19	27.9
**How well do you think the media portrays the ASD problem?**		
Always	14	20.6
Sometimes	33	48.5
Never	19	27.9
Don’t know	2	2.9

### Available services for children with ASD

Table 6 shows services available for ASD care. 97.1% of the ASD children did not have any health insurance, 72.1% received government allowance for ASD, and 48.5% believed that the media plays an important role to highlight the problem of autism.

## Discussion

Due to a lack of awareness in society, parents of children with autism face several obstacles [24]. The study investigated caregivers’ knowledge and practice of children with ASD in Bangladesh. The mean score of knowledge among caregivers was 7.16 (SD = 2.09) out of 12, or 59.67%. A prior study in urban Bangladesh found that almost 95% of parents had little or no understanding of the disorder before their children were diagnosed with autism [25]. A study in Iraq found that approximately 64% of the sample had fair knowledge while 1% had poor knowledge, and the remainder (35%) had good knowledge regarding autism, providing some comparative data from a society less focused on Western medicine [25].

In the present study, 56% of participants indicated that children with ASD had difficulty communicating with others, and 57% had behavioral issues. This would have a knock-on effect; Barker et al. investigated parents with autistic children over a 10-year period, and discovered that when the child’s behavioral problems were more severe, the mothers’ depression symptoms were also more severe [26].

Our study shows that 95.6% of caregivers had no formal training in how to care for children with ASD, and 26.5% of children with ASD were raised by their grandparents or others. It is impossible to stress the importance of parental training and psychoeducation in dispelling parental preconceptions and assisting them in understanding the nature of the condition [27]. Group work interventions can also be beneficial to families, in assisting families in better sharing their feelings and concerns, educating parents on the various ways to cope and handle a specific concern, and helping them in overcoming their sense of isolation [28]. When parents are depressed or appear burned out as a result of their child’s condition, wider family support can be helpful [28].

In this study, most children did not have health insurance (97.1%). In order to provide free physiotherapy and other medical assistance to the disabled and autistic population of the country, experimental disability services, and help centers were set up in five districts of the country in the financial year 2009–2010 [29]. The uptake of these services is not known. All caregivers confirmed that the child had an official diagnosis from a clinical psychologist or genetic test. It is possible that people may not able to continue treatment due to financial difficulties Some families have might not have noticed anything unusual in their children until it was too late [30]. If treatment had been started earlier, it would have been possible to reduce the amount of burden. About 90% of respondents said they were not supported at the structural or community level, nor were they supported by NGOs. Many complications of autism can be avoided by early diagnosis and treatment. Early intervention is important for children with ASD to maximize their health and functional potential. Many children are not diagnosed with ASD until they are school-age or older, which then limits their chances of receiving early intervention [30]. Late diagnosis has been identified as one of the major causes of reduced quality of life. Early intervention can result in significant improvements in cognition, communication, social skills, and adaptive behavior in young children with ASD [31]. Systematic reviews and meta-analyses of randomized trials found that both highly structured, behaviorally based early intervention and naturalistic developmental behavioral interventions have positive effects on many developmental domains for young children with ASD [32].

## Limitations

The present study has some limitations. Firstly, it was a cross-sectional study therefore causal relationships cannot be established. A longitudinal study may be more appropriate in this regard. Secondly, the study was conducted in a specific area of Bangladesh with a small sample size, therefore findings cannot be extrapolated to other areas of Bangladesh or other countries. A future study with a bigger sample size including representative areas is warranted. Finally, the study included limited measures regarding the knowledge and practice of children with ASD. A comprehensive measure should be investigated in the future.

## Conclusions

The findings revealed that caregivers lacked understanding and had some misconceptions about how to treat children with autism. It is intended that by identifying weak points in practice towards children with ASD, better planned educational and behavioral modification efforts can be made to increase knowledge. Pediatricians and other primary health care professionals are in a position to provide important long-term medical care, as well as support, educate, and guide them to observational evidence, in addition to determining ASDs through surveillance and screening, conducting an etiologic evaluation, establishing a diagnosis, and providing genetic counseling after the diagnosis.

## Data Availability

The datasets used and/or analyzed during the current study are available from the corresponding author on reasonable request.

## Abbreviations

ASD: Autism spectrum disorder
BSMMU: Bangabandhu Sheikh Mujib Medical University
